# Screening for Drug and Alcohol Use Disorders and Their Association with HIV-Related Sexual Risk Behaviors among Men Who Have Sex with Men in Peru

**DOI:** 10.1371/journal.pone.0069966

**Published:** 2013-08-06

**Authors:** Kaysia T. Ludford, Panagiotis Vagenas, Javier R. Lama, Jesus Peinado, Pedro Gonzales, Rene Leiva, Monica Pun, Jorge Sanchez, Frederick L. Altice, for the Peruvian HIV Sentinel Surveillance Working Group

**Affiliations:** 1 Yale School of Medicine, Section of Infectious Diseases, AIDS Program, New Haven, Connecticut, United States of America; 2 Asociación Civil Impacta Salud y Educación, Lima, Peru; 3 General Directorate of Epidemiology, Ministry of Health, Lima, Peru; 4 Yale School of Public Health, Department of Epidemiology of Microbial Diseases, New Haven, Connecticut, United States of America; Instituto de Pesquisa Clínica Evandro Chagas/Fundação Oswaldo Cruz, Brazil

## Abstract

**Background:**

Peru's HIV epidemic is concentrated among men who have sex with men (MSM). The contribution of alcohol use disorders (AUDs) to known high-risk behaviors associated with HIV transmission in this context has not been well characterized.

**Methods:**

Between June and October 2011, 5,148 sexually active MSM were recruited using convenience sampling in five cities to participate in a cross-sectional bio-behavioral survey. Five high-risk sexual criteria previously associated with incident HIV infection in this setting were selected *a priori* as the dependent outcomes. Screening for AUDs used the validated Alcohol Use Disorders Identification Test (AUDIT) and AUDS were stratified by severity. Unadjusted and adjusted odds ratios (AOR) were computed to establish the independent correlates of the five dependent outcomes.

**Results:**

The majority (62.8%) of participants met screening criteria for having an AUD, which were independently correlated with each of the following high-risk sexual risk behaviors in the previous 6 months: 1) >5 sexual partners [AOR = 1.76; (1.54–2.02)]; 2) sex with an HIV-infected partner [AOR = 1.29; (1.03–1.62)]; 3) having a sexually transmitted infection [AOR = 1.38; (1.13–1.68)]; 4) being a sex worker [AOR = 1.61; (1.40–1.87)]; and 5) unprotected sex during last encounter [AOR = 1.22; (1.09–1.38)]. Recent drug use was also correlated with having >5 sexual partners [AOR = 1.42 (1.19–1.71)], sex work [AOR = 1.97 (1.63–2.39)] and unprotected sex during last encounter [AOR = 1.31 (1.11–1.54)]. For each dependent variable, the association with AUDs significantly increased with increasing AUD severity.

**Conclusions:**

AUDs are highly prevalent among MSM in Peru and are associated with increased HIV risk-taking behaviors that are associated with HIV transmission. Strategies that target problematic drinking such as medication-assisted therapy, behavioral counseling and structural interventions could potentially reduce risky behaviors and ultimately reduce HIV transmission among MSM in Peru.

## Background

While HIV prevalence in Peru remains low at 0.2%, it underestimates the country's concentrated HIV epidemic among men who have sex with men (MSM) [1]. From 1996 to 2002, HIV prevalence from serosurveillance studies among MSM increased from 18.5% to 22.3% in Lima [2]. HIV infection in this group has been associated with homosexual self-identification, high-risk sexual behaviors, cocaine use before or during sex, and sexually transmitted infections (STI) [3]. Nonetheless, the extent to which these risk behaviors are mediated by the disinhibiting use of alcohol and/or other drugs is not well characterized.

Though many studies assessing the association between alcohol use and HIV risk behaviors have been hampered by the use of non-validated measures of alcohol use and non-specific or non-validated definitions of HIV risk that correlate with HIV incidence, they have nevertheless shown correlations with a variety of risky sexual behaviors [4]–[7]. Specifically, global, situational, and to a lesser extent event-level studies in North America and Africa have demonstrated associations between alcohol use and risky sexual behaviors [8]–[12]. International experts have recently called for more comprehensive services for MSM with or at risk for HIV, including screening for and treatment of substance use disorders [13]. The extent to which alcohol consumption meets screening criteria as a treatable alcohol use disorder (AUD) in most available studies has yet to be examined.

The lack of rigorous screening for having an AUD, a chronic and relapsing condition that can be effectively treated using behavioral and/or pharmacological treatments, and its association with validated high-risk sexual behaviors associated with incident HIV infections has yet to be examined globally or even in the Peruvian context.

## Methods

### Ethics Statement

This research project was approved by the Institutional Review Boards of Impacta, Peru and of Yale University. All procedures were explained to participants, who then read and signed informed consent forms.

### Study participants

Between June and October 2011, 5,575 Peruvian MSM were recruited using modified snowball methods and peer-educator outreach in previously mapped venues frequented by MSM in Lima and four other cities: Ica, Piura, Iquitos and Pucallpa. Sample size was large based on calculations of previous HIV prevalence and incidence data. Eligibility included age ≥18, being genetically male, and self-reporting at least one male sexual partner in the previous 12 months.

### Study procedures

After informed consent procedures and pre-test HIV counseling, participants underwent HIV and syphilis testing followed by a 45-minute computer-assisted self-administered interview (CASI) assessing sexual risk behaviors and alcohol and drug use. Afterwards, they underwent post-test counseling, receipt of test results and treatment using Peruvian national guidelines.

### Variable definitions

The 5 dependent variables, high-risk sexual behaviors associated with incident HIV infection, were decided *a priori* using the Alaska sexual risk criteria for behaviors during the previous 6 months [14]: 1) having a STI; 2) self-identification as a sex worker; 3) no condom use during last anal intercourse; 4) anal intercourse with more than 5 partners; and 5) being a sexual partner of an HIV-infected male.

AUDs were defined using the World Health Organization's validated 10-item Alcohol Use Disorders Identification Test (AUDIT), validated in Spanish, with standard cut-offs to define any AUD (score ≥8). Participants with AUDs, were further divided into severity levels, including alcohol dependence (score ≥20), harmful drinking (score = 17–19) and hazardous drinking (score = 8–16). Though the AUDIT was initially created as a screening instrument, these AUD cut-offs were subsequently validated for their association with a clinically-defined AUD and the scores have high internal consistency, high sensitivity and specificity and positive predictive value [15]. Other independent variables included “sex work”, defined as receiving money, goods or rent in exchange for sex, “low income”, defined as earning less than the local minimum legal wage (750 Soles/month [$283 US/month]) and “drug use” in the three months prior to the study. Drug use was defined if they injected drugs or reported any use of marijuana, “pasta” or powder cocaine, amphetamines, poppers or ecstasy in the previous 3 months. Unlike the timeframe for the dependent variables (past 6 months), the 3-month time period for drug use was selected in order to allow for comparisons with previous Peruvian HIV Sentinel Surveillance studies.

### Data Analyses

Of the 5,575 participants initially recruited, 5,148 (92%) agreed to full participation by completing both HIV testing and the 45-minute behavioral survey. Statistical analyses were performed on this final sample with and complete data using the IBM SPSS Statistics software, version 19. Bivariate logistic regression analyses were conducted to determine associations between each of the 5 dependent high-risk sexual risk behaviors described above with covariates assessing: 1) demographic factors, 2) AUDs and 3) drug use. Covariates significant at p<0.10 were subsequently included in multivariate logistic regression analyses. Non-parametric testing was used for variables that were not normally distributed. Adjusted odds ratios (with 95% confidence intervals) were reported; p<0.05 was deemed statistically significant.

## Results

During the 5-month study period, among the 5,148 final study participants, the majority was below 30 years old, earned low incomes and had at least some secondary schooling. Time constraints were the most common reason for survey non-completion. Table 1 summarizes the characteristics of the 5,148 participants with complete data. With regard to sexual orientation, over half (58%) identified themselves as ‘gay’ with the rest identifying as bisexual (30%) or heterosexual (13%). Using standardized screening measures, nearly two thirds (62.8%) of participants met criteria for having an AUD with most of these being in the hazardous drinking category (AUDIT 8–16). Of those meeting AUD screening criteria, 27% had alcohol dependence (AUDIT ≥20).

**Table 1:** Sociodemographic, alcohol use and sexual behavioral characteristics of the study sample (N = 5148).

Characteristic	N = 5148 (%)
Mean Age (± S.D.^a^)	29.5±9
Monthly income	
None	1192 (23.2)
Less than minimum legal wage^b^	2406 (46.7)
Greater than minimum legal wage	1532 (29.8)
Education	
Primary or less only	832 (16.2)
Some secondary	2484 (48.3)
Some tertiary	1820 (35.4)
Geographic Distribution	
Greater Lima	2725 (52.9)
Iquitos	608 (11.8)
Piura	594 (11.5)
Pucallpa	604 (11.7)
Ica	601 (11.7)
Sexual Orientation (self-defined)	
Homosexual (gay)	2956 (57.4)
Bisexual	1531 (29.7)
Heterosexual	643 (12.5)
Transgender	
Yes	700 (13.6)
No	4427 (86.0)
Sexual Role	
Insertive only	1990 (38.7)
Receptive only	1756 (34.1)
Versatile (both insertive and receptive)	1380 (26.8)
Self-reported sexually transmitted infections (STI) in the prior 12 months	
No	3842 (74.6)
Yes (any)	1289 (25.0)
STI in past 6–12 months	658 (51.0)
STI in past 6 months	631(49.0)
Previously tested for HIV	
Yes	3003 (58.3)
No	2123 (41.2)
Unprotected sex during past sexual encounter	
Yes	2377 (46.2)
No	2749 (53.4)
Any drug use in past 3 months (cocaine, pasta, amphetamines, marijuana, MDMA^c^, poppers)	
Yes	459 (8.9)
No	4337 (84.2)
Alcohol Use Disorders (AUD)	
No AUD or social drinking	1879 (36.5)
Any AUD	3233 (62.8)
Hazardous Use	1905 (58.9)
Harmful Use	468 (14.5)
Dependent Use	860 (26.6)

Percentages of non-missing data are shown.

a S.D.  =  Standard Deviation.

b Minimum legal wage in Peru: 750 Soles/month (US $283/month).

c MDMA = 3,4-methylenedioxy-N-methylamphetamine (Ecstasy).

### Bivariate sex-risk correlates of alcohol use disorders

**Figure 1:**
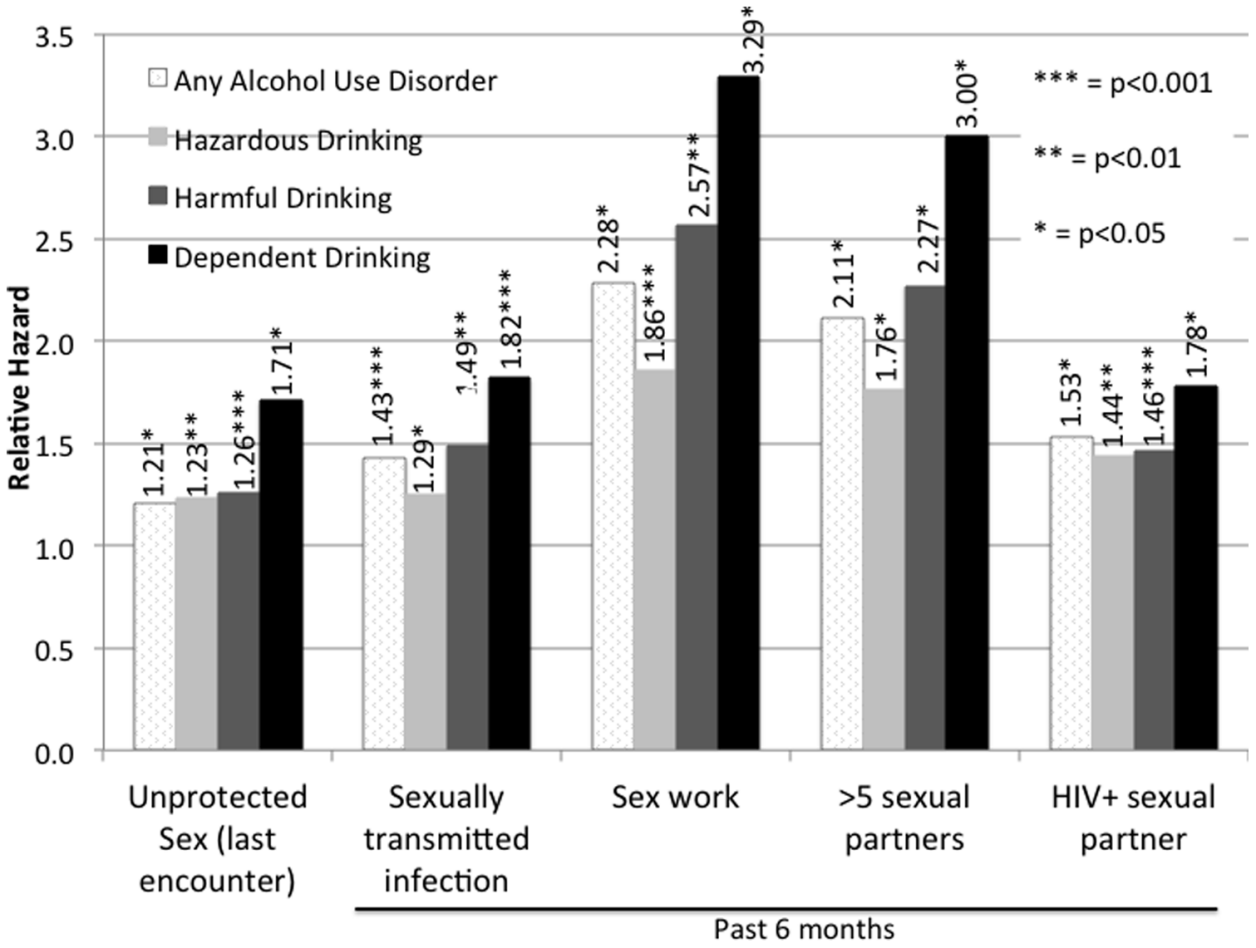
Bivariate correlation between alcohol use disorders and alcohol use severity and high-risk HIV risk behaviors.

Figure 1 depicts the extent to which any AUD (AUDIT ≥8), stratified by hazardous, harmful, or dependent drinking, was associated with each of the five dependent high-risk behaviors in the bivariate analysis. Importantly, having an AUD was independently associated with each of the five high-risk behaviors, with the strongest associations observed for those who had increased AUD severity and: a) were sex workers or b) had >5 male sexual partners in the previous 6 months. Sex workers and participants with multiple partners in the previous six months were more than twice as likely to have an AUD compared to participants who did not engage in sex work or who had fewer sexual partners, respectively. Strikingly, for all 5 dependent variables, the associations with AUDs incrementally increased with higher levels of AUD severity (hazardous<harmful<dependent drinking). For example, participants reporting more than 5 male partners had 1.8, 2.3 and 3.0-fold higher odds of being hazardous, harmful and dependent alcohol drinkers, respectively, compared to those without an AUD.

### Bivariate and Multiple Logistic Regression Associations with High Risk Behaviors

Table S1 provides extensive details for model building through inclusion of bivariate and multivariate modeling for each of the five dependent HIV-related risk behaviors. Specifically, bivariate analysis demonstrated significant correlations between all the dependent variables and AUDs, sex work, bisexual orientation, transgender identity, receptive anal intercourse and living outside Lima. The strongest correlates of having >5 sexual partners were being involved in sex work and having an AUD, while the strongest correlate of having an HIV-infected partner was heterosexual self-identification. Sex workers were more likely to identify as transgendered compared to participants who did not engage in sex work. Sex workers and transgendered participants were less likely to engage in unprotected sex during their last sexual encounter, with unadjusted odds ratios of 0.86 and 0.64 respectively. Though recent drug use was less commonly reported, it also correlated with three of the dependent variables: 1) having >5 sexual partners [AOR = 1.42 (1.19–1.71)]; 2) sex work [AOR = 1.97 (1.63–2.39)]; and 3) unprotected sex during last encounter [AOR = 1.31 (1.11–1.54)].

Table 2 summarizes the final multiple regression findings from Table S1 for each of the 5 high-risk behavior outcomes. After construction of the multivariate logistic regression models, meeting screening criteria for having an AUD remained the only covariate significantly associated with all 5 dependent high-risk behaviors. Recreational drug use independently correlated with three of the five high-risk behaviors and was most strongly associated with being a sex worker.

**Table 2:** Correlates of HIV-related risk behaviors associated with HIV transmission (N = 5,148).

Covariates	Adjusted Odd Ratios (95% Confidence Intervals) for dependent variables				
	>5 sexual partners	HIV-infected partner	STI	Sex work	Unprotected sex (last encounter)
Any alcohol use disorder	**1.76 (1.54–2.02)** ***	1.29 (1.03–1.62)*	1.38 (1.13–1.68)**	**1.61 (1.40–1.87)** ***	**1.22 (1.09–1.38)** ***
Any recent drug use	**1.42 (1.19–1.71)** ***	1.04 (0.80–1.36)	-γ	**1.97 (1.63–2.39)** ***	**1.31 (1.11–1.54)** ***
Client of sex worker	**1.54 (1.29–1.84)** ***	1.33 (1.03–1.74)*	1.15 (0.91–1.46)	**2.90 (2.41–3.50)** ***	-γ
Salary above minimum wage	**1.43 (1.25–1.64)** ***	**1.45 (1.17–1.79)** ***	1.23 (1.02–1.49)*	0.91 (0.78–1.06)	-γ
Bisexual or heterosexual	0.85 (0.72–1.01)	**2.41 (1.85–3.14)** ***	0.90 (0.72–1.14)	**1.79 (1.53–2.09)** ***	0.90 (0.78–1.04)
Transgender	**1.47 (1.21–1.78)** ***	0.77 (0.52–1.13)	-γ	**4.0 (3.27–4.89)** ***	**0.72 (0.60–0.86)** ***
Receptive/versatile sexual role	**1.34 (1.14–1.58)** ***	0.83 (0.64–1.06)	1.32 (1.04–1.67)*	-γ	**0.71 (0.61–0.82)** ***
Resident of Lima (capital)	**0.80 (0.71–0.91)** ***	0.87 (0.71–1.07)	**0.61 (0.51–0.73)** ***	**0.65 (0.57–0.75)** ***	0.85 (0.76–0.96)**
>30 years old	-γ	-γ	**0.72 (0.59–0.88)** ***	**0.61 (0.52–0.71)** ***	0.9 (0.8–1.01)
>5 sexual partners	N/A	-γ	1.30 (1.08–1.57)**	**2.71 (2.36–3.12)** ***	-γ
HIV-infected partner	-γ	N/A	-γ	1.37 (1.10–1.72)**	-γ
STI	1.30 (1.08–1.57)**	-γ	N/A	1.32 (1.08–1.62)**	-γ
Sex work	2.67 (2.33–3.07) **	1.38 (1.10–1.72)**	1.31 (1.08–1.60)*	N/A	**0.81 (0.72–0.92)** ***
Unprotected sex (last encounter)	-γ	-γ	-γ	0.81 (0.70–0.93)**	N/A

*** p<0.001; **p<0.01; *p-value<0.05.

γ Was not included in multivariate analysis.

## Discussion

To our knowledge, this study is the first to examine the prevalence of AUDs among Peruvian MSM using the AUDIT, which has been internationally validated for distinguishing between the various levels of severity of AUDs and assessed them in relationship to risk behaviors associated with HIV transmission. Our study confirms findings from a recent cross-sectional study of Peruvian MSM that documents a high prevalence of alcohol-related problems, but which used the CAGE questionnaire. Their study, unlike ours, found problem drinking to be associated with previous sexual coercion, alcohol consumption during recent sex, sex work and self-reported HIV+ status [6]. In that study, unlike ours, they assessed the correlates of “problematic drinking” and not the association of alcohol consumption specifically on HIV risk behaviors associated with HIV transmission.

Though AUDs vary in severity, our assessment that deployed an internationally-validated screening instrument demonstrates that the prevalence of AUDs is extremely high in this population with nearly two thirds (62.8%) of participants meeting screening criteria for having an AUD. Over a third (37%) met criteria for hazardous drinking (binge drinking), which despite being the least severe type of AUD, remains highly problematic because of the increased likelihood for progression over time to harmful drinking or alcohol dependence. Hazardous drinking necessitates, at a minimum, counseling with the recommendation to reduce drinking linked to provision of educational materials that not only provide information about alcohol's potential for harm to the patient and to others, but motivation to reduce heavy drinking due to the increased likelihood of progressing toward more severe AUDs. For HIV prevention purposes, reducing the progression to alcohol dependence, or more severe drinking, would likely be associated with decreased HIV risk behaviors since heavy drinking days are associated with increased risk-taking [16]. Those meeting screening criteria for harmful drinking should likewise undergo counseling content similar to that for hazardous drinking, but would also require additionally monitoring and ongoing counseling. The interventions for hazardous and harmful drinking can be brief and may take various forms, ranging from providing 5 minutes of counseling about cutting down on drinking, to several brief sessions, depending on the severity of alcohol consumption and its effect on health, legal or social problems. At a minimum, the AUDIT creators suggest that the content of counseling sessions: (i) present screening results; (ii) identify risks and discuss consequences; (iii) provide medical advice; (iv) solicit patient commitment; (v) identify specific goals, specifically reduced drinking or abstinence; and (vi) give advice and encouragement [17].

Alcohol intoxication and drug use increases sexual risk behaviors via various mechanisms including drinking environments, expectancies about its sexual enhancement, its neurocognitive effects and associated poor decision-making [12]. Within MSM communities, the association between alcohol and/or drug use and sex may be even more pronounced for a number of reasons. First, gay bars have historically been among the few safe gathering spaces for MSM, functioning in some cases as “*de facto* community centers” [18]. Second, disinhibiting substances may help overcome the taboo associated with stigmatizing behaviors. Finally, men whose first MSM sexual encounter that involved alcohol or drugs are more likely to continue using them in subsequent sexual encounters [18]. Equally disturbing is the high proportion of participants (16.7%) meeting screening criteria for alcohol dependence, over 50% higher than the 10% prevalence reported in the general Peruvian population in 2002, using the 9^th^ Edition of the World Health Organization's International Classification of Diseases criteria (ICD-9) for alcohol dependence [19].

Central to our study was that the presence of AUDs was significantly associated with all five high risk behaviors assessed in our survey, based on the Alaska sexual risk criteria, that have been associated with HIV transmission [14]. Specifically, AUDs were associated with: 1) having more than five sexual partners; 2) unprotected sex during last sexual encounter; 3) having a STI; 4) commercial sex work; and 5) sex with an HIV-infected partner – in the previous six months. Moreover, there was an increasing linear association between AUD severity level and all of these outcomes.

The association of high-risk sexual behaviors and AUDs has important implications for HIV prevention. Higher levels of alcohol consumption, beyond “sensible drinking” criteria, is associated with social and sexual disinhibition, including decreased condom use [20]–[23]. Effective treatment of AUDs would likely significantly reduce sexual risk behaviors and ultimately HIV transmission. A number of studies have shown that behavioral interventions to reduce alcohol consumption alone have a small or negligible effect; the most effective method to treat AUDs is treatment with medication-assisted therapies [24]. Though it is seductive to speculate that the disinhibiting effects of alcohol directly cause the high risk-taking behaviors observed in this sample, but the cross-sectional nature of this study only allows us to examine the strength of such associations without proving causality. Event-level granularity that establishes concurrency between alcohol use and risky behaviors, however, is needed to determine if it is the disinhibiting effects of alcohol or, alternatively, unique personality traits, that directly contribute to increased sexual risk [25], [26]. Specifically, personality traits associated with sexual adventurism have been independently associated with lack of condom use and alcohol abuse, but not all individuals who are sexually adventurous use alcohol or drugs [27], [28]. Disentangling causality would therefore better inform interventions for MSM because the type of intervention needed would differ significantly between those whose sexual risk is preceded by alcohol use versus others whose personality traits lead to sexual adventurism [25].

The independent association between AUDs and having had a recent STI is of interest. The presence of STIs, especially ulcerative ones, facilitates HIV transmission [29]. Since the presence of STIs is an indicator of high-risk behaviors in general [30] and AUDs in our sample were associated with recent STIs, there is an urgent need to screen simultaneously for AUDs and acute HIV infection (AHI) among MSM presenting to STI clinics. This is particularly relevant since AHI is associated with high levels of onward HIV transmission since viral replication is inordinately high [31] and the presence of AUDs is associated with disinhibition and sexual risk.

Interestingly, having an AUD was one of the strongest correlates for engaging in sex work. Among the transgendered community in Peru, many transgendered women are involved in sex work [32]. Many sex workers admit to consuming increased levels of alcohol during their sexual encounters with clients, upon the insistence of their clients. Additionally sex workers often recruit clients in nightlife venues where drinking is prevalent. The more time a sex worker spends in such venues, the more likely they are to engage clients. Increased time in these settings, however, potentially results in increased time consuming alcohol [33].

Independent from having an AUD, drug use was associated with three of the five high-risk HIV behaviors, with the exception of having recently had a STIs or sex with a known HIV-infected partner. These data are consistent with prior studies documenting a relationship between drug use and risky sex among MSM [26], [34]. It should be noted, however, that the prevalence of drug use by participants in this study was 6 times lower than the prevalence of AUDs. In our study, less than 9% of subjects had used drugs, primarily marijuana and inhaled, sniffed and “pasta” (free-based” cocaine). These data are distinct from North American MSM, who report high levels of drug use, especially club drugs that are associated with high HIV risk behaviors [35]. Our findings confirm the high prevalence of alcohol use from a recent trial that included MSM from Peru and the U.S. where 52% of Peruvian MSM reported high levels of alcohol use during sex while only 5% reported concomitant drug use. Among U.S. MSM in the same study, equal proportions reported drug and alcohol use (39% and 38%, respectively) [36]. So, while it is important to design risk reduction intervention strategies aimed at lowering drug use, such strategies should be specifically targeted at the small subset of MSM that use drugs. Problematic alcohol use, on the other hand, is more widespread among MSM and in addition to individual-level interventions, should simultaneously be addressed as part of national HIV prevention interventions, including structural interventions that target the setting [37].

In light of the associations between AUDs and sexual risk behaviors, HIV risk reduction interventions should be developed to address not only sexual behavioral risks, but also alcohol consumption. Whereas current HIV prevention strategies do not ignore alcohol problems, most address it in a limited manner. It is therefore possible that problematic alcohol use negatively interferes with current evidence-based HIV prevention interventions that don't address alcohol problems. A multisite HIV Prevention Trials Network study that aimed to address alcohol use in the context of HIV risk behavior found that participants who showed signs of problematic alcohol use one year following the completion of alcohol use reduction interventions were more likely to relapse to having unprotected sex with multiple partners compared with those who remained completely alcohol-free after completing the intervention [38].

Given that a significant proportion of participants scored 20 or higher on the AUDIT, suggestive of meeting criteria for alcohol dependence, they should be referred to a specialist for diagnostic evaluation and treatment for dependent alcohol use, which can be effectively treated using evidence-based practices. Three medications are approved for the treatment of AUDs, including disulfiram, acamprosate [24] and both the oral and extended-release formulation of naltrexone. Disulfuram, through inhibition of aldehyde dehydrogenase, causes acute sickness (e.g., aversion therapy through flushing, nausea, headache, sweating, weakness, increased blood pressure) when alcohol is ingested and does not reduce craving. It should be reserved only for highly motivated patients whose medication adherence is strictly observed. Both acamprosate and naltrexone reduce craving. In a large multisite trial, however, the use of naltrexone was superior to acamprosate either alone or in combination with weekly cognitive behavioral counseling sessions or combined with acamprosate [39]. Naltrexone reduces relapse to heavy alcohol use by blocking opioid receptors in the mesolimbic reward pathway that become activated by ethanol such that the pleasure-response associated with drinking (“the buzz”) is blunted. Alternatively, acamprosate works through a different mechanism involving modulation and/or normalization of alcohol-disrupted brain activity, particularly in the GABA and glutamate neurotransmitter systems. If naltrexone proves to be effective at lowering the levels of heavy drinking days among Peruvian MSM, it has the potential to reduce HIV risk behaviors as well as improve ART adherence for HIV-infected individuals with AUDs, and ultimately decreasing community-wide HIV transmission [4].

Though the findings from this study have important implications for HIV prevention among Peruvian MSM with AUDs, and perhaps all MSM with AUDs, there are some methodological limitations. Convenience samples recruited through a non-statistically determined referral chain mechanism may result in a non-representative sample of Peruvian MSM. Though the large sample size, multisite recruitment of this sample and similar findings from a previous population-based study showing that the majority of MSM (over 90%) had been to a gay bar in the previous 6 months [20] may, in part, decrease these concerns, it is critical to note that our sample is not necessarily representative of all MSM. Our study was cross-sectional, only allowing us to demonstrate high correlation, but not causality. Last, our study relied on self-report for alcohol and drug use and sexual risk behaviors. While concerns about social desirability response for culturally stigmatizing drug and alcohol use and sexual risk behaviors may result in under-reporting, the use of the anonymous CASI assessment and the remarkably high responses to these questions reduce these concerns. Notwithstanding these limitations, the large sample size and consistent and strong correlation between AUDs and drug use and validated sexual risk behaviors associated with HIV transmission suggest a relationship that is worthy of future intervention.

## Conclusions

The prevalence of AUDs is high among MSM in Peru and associated with increased levels of HIV risk-taking behaviors. The increasing severity of AUDs is associated with the increasing likelihood that MSM engage in high-risk behaviors that can result in HIV transmission. These findings may, in part, explain the continued concentrated HIV epidemic among MSM and have important implications for HIV prevention, diagnosis and entry into and retention in care. These individuals would greatly benefit from strategies that reduce problematic drinking, including medication-assisted therapy, behavioral counseling and structural interventions.

## Supporting Information

Table S1 **Correlates of HIV-related risk behaviors associated with HIV transmission.**
(DOCX)
