# Supplementary material for: Screening for Drug and Alcohol Use Disorders and Their Association with HIV-Related Sexual Risk Behaviors among Men Who Have Sex with Men in Peru
Source: PLoS One. 2013 Aug 6;8(8):e69966. doi: 10.1371/journal.pone.0069966 (PMC3735581; doi:10.1371/journal.pone.0069966)
Supplement: Table S1 — Correlates of HIV-related risk behaviors associated with HIV transmission. (DOCX) [file pone.0069966.s001.docx]

**Table S1: Correlates of HIV-related risk behaviors associated with HIV transmission**

1A. Correlates of having more than 5 sexual partners in the past six months

| **Covariates** | **Bivariate** | | **Multivariate** | |
| --- | --- | --- | --- | --- |
|  | Unadjusted Odds Ratio (95% CI) | P-value | Adjusted Odds Ratio (95% CI) | P-value |
| Sexually transmitted infection (past 6 months) |  |  |  |  |
| No | Referent |  |  |  |
| Yes | 1.58 (1.33-1.88) | <0.001 | 1.30 (1.08-1.57) | 0.006 |
| Sex Work (past 6 months) |  |  |  |  |
| No | Referent |  |  |  |
| Yes | 3.38 (2.98-3.84) | <0.001 | 2.67 (2.33-3.07) | <0.001 |
| Any alcohol use disorder |  |  |  |  |
| No | Referent |  |  |  |
| Yes | 2.11 (1.86-2.39) | <0.001 | 1.76 (1.54-2.02) | <0.001 |
| Client of a sex worker (past 6 months) |  |  |  |  |
| No | Referent |  |  |  |
| Yes | 2.17 (1.84-2.55) | <0.001 | 1.54 (1.29-1.84) | <0.001 |
| Any drug use (pasta or powder cocaine, amphetamines, marijuana, MDMA, poppers) |  |  |  |  |
| No | Referent |  |  |  |
| Yes | 1.93 (1.64-2.26) | <0.001 | 1.42 (1.19-1.71) | <0.001 |
| Salary |  |  |  |  |
| Below minimum wage | Referent |  |  |  |
| Above minimum wage | 1.19 (1.03-1.38) | 0.017 | 1.43 (1.25-1.64) | <0.001 |
| Sexual orientation |  |  |  |  |
| Homosexual (gay) | Referent |  |  |  |
| Bisexual or Heterosexual | 0.75 (0.66-0.84) | <0.001 | 0.85(0.72-1.01) | 0.057 |
| Transgender |  |  |  |  |
| No | Referent |  |  |  |
| Yes | 2.28(1.93-2.70) | <0.001 | 1.47 (1.21-1.78) | <0.001 |
| Predominant sexual role |  |  |  |  |
| Insertive only | Referent |  |  |  |
| Receptive or versatile | 1.53 (1.36-1.73) | <0.001 | 1.34 (1.14-1.58) | <0.001 |
| Geographic Location |  |  |  |  |
| Outside Lima | Referent |  |  |  |
| Lima | 1.47 (1.31-1.67) | <0.001 | 0.80 (0.71-0.91) | 0.001 |
| Living arrangement |  |  |  |  |
| Alone | Referent |  |  |  |
| With others | 0.83 (0.72-0.96) | 0.011 | 0.91 (0.78-1.06) | 0.23 |
| Age |  |  |  |  |
| 30 years old and below | Referent |  |  |  |
| Over 30 years old | 1.01 (0.90-1.14) | 0.839 | - | - |

1B. Correlates of having had a HIV-infected partner in the past 6 months

| **Covariates** | **Bivariate** | | **Multivariate** | |
| --- | --- | --- | --- | --- |
|  | Unadjusted Odds Ratio (95% CI) | P-value | Adjusted Odds Ratio (95% CI) | P-value |
| Sexually transmitted infection (past 6 months) |  |  |  |  |
| No | Referent |  |  |  |
| Yes | 1.33 (1.02-1.73) | 0.04 | 1.24 (0.93-1.65) | 0.14 |
| Sex Work (past 6 months) |  |  |  |  |
| No | Referent |  |  |  |
| Yes | 1.51 (1.25-1.83) | <0.001 | 1.38 (1.10-1.72) | 0.005 |
| Any alcohol use disorder |  |  |  |  |
| No | Referent |  |  |  |
| Yes | 1.53 (1.25-1.88) | <0.001 | 1.29 (1.03-1.62) | 0.029 |
| Client of a sex worker (past 6 months) |  |  |  |  |
| No | Referent |  |  |  |
| Yes | 1.35 (1.06-1.72) | 0.015 | 1.33 (1.03-1.74) | 0.03 |
| Any drug use (pasta or powder cocaine, amphetamines, marijuana, MDMA, poppers) |  |  |  |  |
| No | Referent |  |  |  |
| Yes | 1.58 (1.25-1.99) | <0.001 | 1.04 (0.80-1.36) | 0.75 |
| Salary |  |  |  |  |
| Below mínimum wage | Referent |  |  |  |
| Above mínimum wage | 1.32 (1.06-1.64) | 0.014 | 1.45 (1.17-1.79) | 0.001 |
| Sexual orientation |  |  |  |  |
| Homosexual | Referent |  |  |  |
| Bisexual or Heterosexual | 2.93 (2.39-3.50) | <0.001 | 2.41 (1.85-3.14) | <0.001 |
| Transgender |  |  |  |  |
| No | Referent |  |  |  |
| Yes | 0.47(0.33-0.66) | <0.001 | 0.77(0.52-1.13) | 0.18 |
| Predominant sexual role |  |  |  |  |
| Insertive only | Referent |  |  |  |
| Receptive or versatile | 0.47 (0.39-0.57) | <0.001 | 0.83 (0.64-1.06) | 0.14 |
| Geographic Location |  |  |  |  |
| Outside Lima | Referent |  |  |  |
| Lima | 0.82 (0.68-0.99) | 0.038 | 0.87 (0.71-1.07) | 0.19 |
| Living arrangement |  |  |  |  |
| Alone | Referent |  |  |  |
| With others | 1.31 (1.03-1.68) | 0.03 | 1.24 (0.95-1.62) | 0.11 |
| Age |  |  |  |  |
| 30 years old and younger | Referent |  |  |  |
| Over 30 years old | 0.87 (0.72-1.07) | 0.184 | - | - |

1C. Correlates of having had a STI in the past 6 months

| **Covariates** | **Bivariate** | | **Multivariate** | |
| --- | --- | --- | --- | --- |
|  | Unadjusted Odds Ratio (95% CI) | P-value | Adjusted Odds Ratio (95% CI) | P-value |
| Five or more sexual partners (past 6 months) |  |  |  |  |
| No | Referent |  |  |  |
| Yes | 1.58 (1.33-1.88) | <0.001 | 1.30 (1.08-1.57) | 0.006 |
| Sex Work (past 6 months) |  |  |  |  |
| No | Referent |  |  |  |
| Yes | 1.56 (1.31-1.85) | <0.001 | 1.31 (1.08-1.60) | 0.02 |
| Any alcohol use disorder |  |  |  |  |
| No | Referent |  |  |  |
| Yes | 1.43 (1.19-1.71) | <0.001 | 1.38 (1.13-1.68) | 0.002 |
| Client of a sex worker (past 6 months) |  |  |  |  |
| No | Referent |  |  |  |
| Yes | 1.38 (1.11-1.71) | 0.004 | 1.15 (0.91-1.46) | 0.25 |
| Salary |  |  |  |  |
| Below minimum wage | Referent |  |  |  |
| Above minimum wage | 1.34 (1.10-1.64) | 0.004 | 1.23 (1.02-1.49) | 0.03 |
| Sexual orientation |  |  |  |  |
| Homosexual | Referent |  |  |  |
| Bisexual or Heterosexual | 0.83 (0.70-0.98) | 0.032 | 0.90 (0.72-1.14) | 0.40 |
| Predominant sexual role |  |  |  |  |
| Insertive only | Referent |  |  |  |
| Receptive or versatile | 1.40 (1.17-1.67) | <0.001 | 1.32 (1.04-1.67) | 0.02 |
| Age |  |  |  |  |
| 30 years old and younger | Referent |  |  |  |
| Over 30 years old | 0.75 (0.62-0.90) | 0.002 | 0.72 (0.59-0.88) | 0.001 |
| Geographic Location |  |  |  |  |
| Outside Lima | Referent |  |  |  |
| Lima | 0.52 (0.40-0.62) | <0.001 | 0.61 (0.51-0.73) | <0.001 |

1D. Correlates of sex work in the past 6 months

| **Covariates** | **Bivariate** | | **Multivariate** | |
| --- | --- | --- | --- | --- |
|  | Unadjusted Odds Ratio (95% CI) | P-value | Adjusted Odds Ratio (95% CI) | P-value |
| More than 5 sexual partners (past 6 months) |  |  |  |  |
| No | Referent |  |  |  |
| Yes | 3.38 (2.98-3.84) | <0.001 | 2.71 (2.36-3.12) | <0.001 |
| Any sexually transmitted infection (past 6 months) |  |  |  |  |
| No | Referent |  |  |  |
| Yes | 1.56 (1.32-1.85) | <0.001 | 1.32 (1.08-1.62) | 0.007 |
| HIV-infected partner (past 6 months) |  |  |  |  |
| No | Referent |  |  |  |
| Yes | 1.51 (1.25-1.83) | <0.001 | 1.37 (1.10-1.72) | 0.005 |
| Unprotected sex (past sexual encounter) |  |  |  |  |
| No | Referent |  |  |  |
| Yes | 0.86 (0.76-0.96) | 0.01 | 0.81 (0.70-0.93) | 0.002 |
| Any alcohol use disorder |  |  |  |  |
| No | Referent |  |  |  |
| Yes | 2.28 (2.01-2.60) | <0.001 | 1.61 (1.40-1.87) | <0.001 |
| Client of a sex worker (past 6 months) |  |  |  |  |
| No | Referent |  |  |  |
| Yes | 2.96 (2.53-3.46) | <0.001 | 2.90 (2.41-3.50) | <0.001 |
| Any drug use (pasta or powder cocaine, amphetamines, marijuana, MDMA, poppers) |  |  |  |  |
| No | Referent |  |  |  |
| Yes | 2.87 (2.46-3.35) | <0.001 | 1.97 (1.63-2.39) | <0.001 |
| Salary |  |  |  |  |
| Below mínimum wage | Referent |  |  |  |
| Above mínimum wage | 0.83 (0.72-0.96) | 0.012 | 0.91 (0.78-1.06) | 0.22 |
| Sexual orientation |  |  |  |  |
| Homosexual | Referent |  |  |  |
| Bisexual or Heterosexual | 1.23 (1.10-1.39) | <0.004 | 1.79(1.53-2.09) | <0.001 |
| Transgender |  |  |  |  |
| No | Referent |  |  |  |
| Yes | 3.29(2.79-3.87) | <0.001 | 4.0 (3.27-4.89) | <0.001 |
| Age |  |  |  |  |
| 30 years old and younger | Referent |  |  |  |
| Over 30 years old | 0.67 (0.59-0.76) | <0.001 | 0.61 (0.52-0.71) | <0.001 |
| Geographic Location |  |  |  |  |
| Outside Lima | Referent |  |  |  |
| Lima | 0.58 (0.52-0.65) | <0.001 | 0.65 (0.57-0.75) | <0.001 |
| Living arrangement |  |  |  |  |
| Alone | Referent |  |  |  |
| With others | 0.84 (0.73-0.96) | 0.012 | 0.90 (0.76-1.08) | 0.25 |
| Education |  |  |  |  |
| Primary only | Referent |  |  |  |
| Secondary or above | 0.81 (0.69-0.94) | 0.006 | 1.00 (0.83-1.21) | 1.0 |

1E. Correlates of unprotected sex (last encounter)

| **Covariates** | **Bivariate** | | **Multivariate** | |
| --- | --- | --- | --- | --- |
|  | Unadjusted Odds Ratio (95% CI) | P-value | Adjusted Odds Ratio (95% CI) | P-value |
|  |  |  |  |  |
| Sex Work (past 6 months) |  |  |  |  |
| No | Referent |  |  |  |
| Yes | 0.86 (0.76-0.96) | 0.01 | 0.81 (0.72-0.92) | 0.001 |
| Any alcohol use disorder |  |  |  |  |
| No | Referent |  |  |  |
| Yes | 1.21 (1.08-1.36) | 0.001 | 1.22 (1.09-1.38) | 0.001 |
| Any drug use (pasta or powder cocaine, amphetamines, marijuana, MDMA, poppers) |  |  |  |  |
| No | Referent |  |  |  |
| Yes | 1.38 (1.18-1.61) | <0.001 | 1.31 (1.11-1.54) | 0.001 |
| Sexual orientation |  |  |  |  |
| Homosexual | Referent |  |  |  |
| Bisexual or Heterosexual | 1.23 (1.10-1.37) | <0.001 | 0.90 (0.78-1.04) | 0.15 |
| Transgender |  |  |  |  |
| No | Referent |  |  |  |
| Yes | 0.64(0.54-0.75) | <0.001 | 0.72 (0.60-0.86) | <0.001 |
| Predominant sexual role |  |  |  |  |
| Insertive only | Referent |  |  |  |
| Receptive or versatile | 0.69 (0.62-0.77) | <0.001 | 0.71 (0.61-0.82) | <0.001 |
| Age |  |  |  |  |
| 30 years old and younger | Referent |  |  |  |
| Over 30 years old | 0.89 (0.79-0.99) | 0.039 | 0.9 (0.8-1.01) | 0.08 |
| Geographic Location |  |  |  |  |
| Outside Lima | Referent |  |  |  |
| Lima | 0.89 (0.79-0.99) | 0.035 | 0.85 (0.76-0.96) | 0.006 |
